# Atrial fibrillation/atrial flutter and reduced volumes of the hippocampus, amygdala, and thalamus: evidence from a community-based MRI study

**DOI:** 10.3389/fnagi.2026.1848538

**Published:** 2026-07-17

**Authors:** Ayano Shima, Moeko Noguchi-Shinohara, Yuta Usui, Yasuko Tatewaki, Benjamin Thyreau, Jun Hata, Tomoyuki Ohara, Takanori Honda, Yasuyuki Taki, Tatsuya Mikami, Tetsuya Maeda, Masaru Mimura, Kenji Nakashima, Jun-ichi Iga, Minoru Takebayashi, Toshiharu Ninomiya, Kenjiro Ono

**Affiliations:** 1 Department of Neurology, Kanazawa University Graduate School of Medical Sciences, Kanazawa, Japan; 2 Department of Aging Research and Geriatric Medicine, Institute of Development, Aging and Cancer, Tohoku University, Sendai, Japan; 3 Department of Health Care Administration and Management, Graduate School of Medical Sciences, Kyushu University, Fukuoka, Japan; 4 Center for Cohort Studies, Graduate School of Medical Sciences, Kyushu University, Fukuoka, Japan; 5 Department of Epidemiology and Public Health, Graduate School of Medical Sciences, Kyushu University, Fukuoka, Japan; 6 Department of Neuropsychiatry, Graduate School of Medical Sciences, Kyushu University, Fukuoka, Japan; 7 Department of Preemptive Medicine, Innovation Center for Health Promotion, Graduate School of Medicine, Hirosaki University, Aomori, Japan; 8 Division of Neurology and Gerontology, Department of Internal Medicine, School of Medicine, Iwate Medical University, Iwate, Japan; 9 Department of Neuropsychiatry, Keio University School of Medicine, Tokyo, Japan; 10 National Hospital Organization, Matsue Medical Center, Matsue, Japan; 11 Department of Neuropsychiatry, Ehime University Graduate School of Medicine, Ehime University, Ehime, Japan; 12 Department of Psychiatry and Neuroscience, Center for Metabolic Regulation of Healthy Aging, Faculty of Life Sciences, Kumamoto University, Kumamoto, Japan

**Keywords:** atrial fibrillation, atrial flutter, brain volume, community-based study, hippocampus

## Abstract

**Introduction:**

Atrial fibrillation (AF) is associated with cognitive decline and dementia even in the absence of clinical stroke, potentially through mechanisms such as silent infarction, small-vessel disease, hypoperfusion, and systemic inflammation. However, most previous studies have focused on global brain measures, and little is known about region-specific structural alterations, particularly within deep gray-matter structures and hippocampal subfields. Evidence regarding atrial flutter (AFL) remains limited. This study aimed to investigate the association between AF/AFL and regional brain and hippocampal subfield volumes in cognitively normal older adults.

**Materials and methods:**

This cross-sectional analysis included 7,074 community-dwelling participants aged ≥65 years from the Japan Prospective Studies Collaboration for Aging and Dementia (JPSC-AD). Structural magnetic resonance imaging with 3D T1-weighted images was processed using FreeSurfer 7.0. Volumes of the cortex, cortical white matter, subcortical gray matter, eight regional brain areas, and hippocampal subfields were evaluated. AF/AFL was identified using electrocardiography. Adjusted mean regional brain volumes and 95% confidence intervals (CIs) were estimated using analysis of covariance. The false discovery rate was controlled using the Benjamini–Hochberg method. Additional exploratory analyses based on self-reported AF history were also performed. Stratified analyses were performed by age (<75 vs. ≥75 years) and sex.

**Results:**

After multivariable adjustment, AF/AFL (171 participants, 2.4%) was associated with smaller volumes of the cortex, cortical white matter, and subcortical gray matter. Among regional structures, AF/AFL was significantly associated with smaller hippocampal and amygdala volumes. At the hippocampal subfield level, reduced fimbria, dentate gyrus, CA1, and subiculum volumes were observed. Analyses based on self-reported history of AF did not demonstrate significant associations with any brain regions. In stratified analyses, participants aged <75 years showed a trend toward smaller hippocampal-tail volume with a significant interaction effect, whereas women exhibited smaller hippocampal volume without a significant sex interaction.

**Discussion:**

In this large community-based sample of cognitively normal older adults, AF/AFL was associated with widespread structural brain alterations and selective vulnerability in memory-related regions, including the hippocampus and amygdala, as well as specific hippocampal subfields. These findings suggest that AF/AFL may be associated with region-specific brain vulnerability before the onset of clinically apparent cognitive impairment.

## 1 Introduction

Atrial fibrillation (AF) is the most common cardiac arrhythmia, and its prevalence increases with age. In addition to its well-established association with ischemic stroke and heart failure, growing evidence indicates that AF is also linked to cognitive decline and dementia. A meta-analysis demonstrated that AF is associated with an increased risk of cognitive impairment and dementia, even in the absence of clinically overt stroke (Kalantarian et al., 2013). Several epidemiological and review studies have proposed that multiple pathophysiological mechanisms–such as silent cerebral infarction, small vessel disease, white-matter lesions, chronic cerebral hypoperfusion, and inflammation–may contribute to AF-related chronic brain injury (Alonso and Arenas de Larriva, 2016). Moreover, population-based and large prospective cohort studies have reported that the association between AF and dementia is more pronounced among women and individuals with earlier onset of AF, suggesting that longer exposure to AF may promote neurodegenerative processes (Ott et al., 1997; Zhang et al., 2023).

Despite these findings, most studies have focused on global structural measures, such as total brain volume, cortical and white matter volumes, or total hippocampal volume. Relatively few investigations have examined region-specific structural alterations, particularly within deep gray-matter structures or hippocampal subfields. Studies evaluating hippocampal subfield volumes in relation to AF are especially scarce, and it remains unclear which subregions are preferentially affected. The hippocampus is not a homogeneous structure but consists of multiple anatomically and functionally distinct subfields, including CA1, the dentate gyrus, and the subiculum, each of which shows different patterns of vascular supply, neuronal vulnerability, and functional specialization (Bartsch et al., 2011; Duvernoy et al., 2013; Kitchigina et al., 2022; Matsumoto et al., 2019; Morris et al., 1990; Sasaki et al., 2018; van Dijk and Fenton, 2018). Previous neuroimaging and experimental studies have demonstrated that specific hippocampal subfields exhibit differential susceptibility to vascular injury, hypoperfusion, and neurodegenerative processes, particularly the selective vulnerability of CA1 neurons to ischemic injury (Bartsch et al., 2015). Therefore, examining hippocampal subfields may provide more precise insights into region-specific brain vulnerability and may help clarify potential mechanisms linking AF/AFL to structural brain alterations that cannot be detected by analyses limited to total hippocampal volume. Accordingly, in the present study, we examined region-specific brain atrophy in relation to AF and AFL in greater detail.

The Japan Prospective Studies Collaboration for Aging and Dementia (JPSC-AD) is an ongoing community-based observational study conducted at eight research sites in Japan. Approximately 10,000 older participants underwent brain magnetic resonance imaging (MRI) and baseline glycemic measures (Ninomiya et al., 2020). Using this large, harmonized, population-based imaging cohort, we investigated structural brain alterations associated with AF and AFL. Automated volumetric segmentation using FreeSurfer was applied to quantify the volumes of the cortex, cortical white matter, and deep gray matter, as well as detailed subregional volumes in structures demonstrating significant associations. When differences in total hippocampal volume were identified, hippocampal subfield volumes were further examined.

This study aimed to clarify the association between AF/AFL and region-specific brain structural alterations in cognitively normal community-dwelling older adults.

## 2 Materials and methods

### 2.1 Study population

**FIGURE 1:**
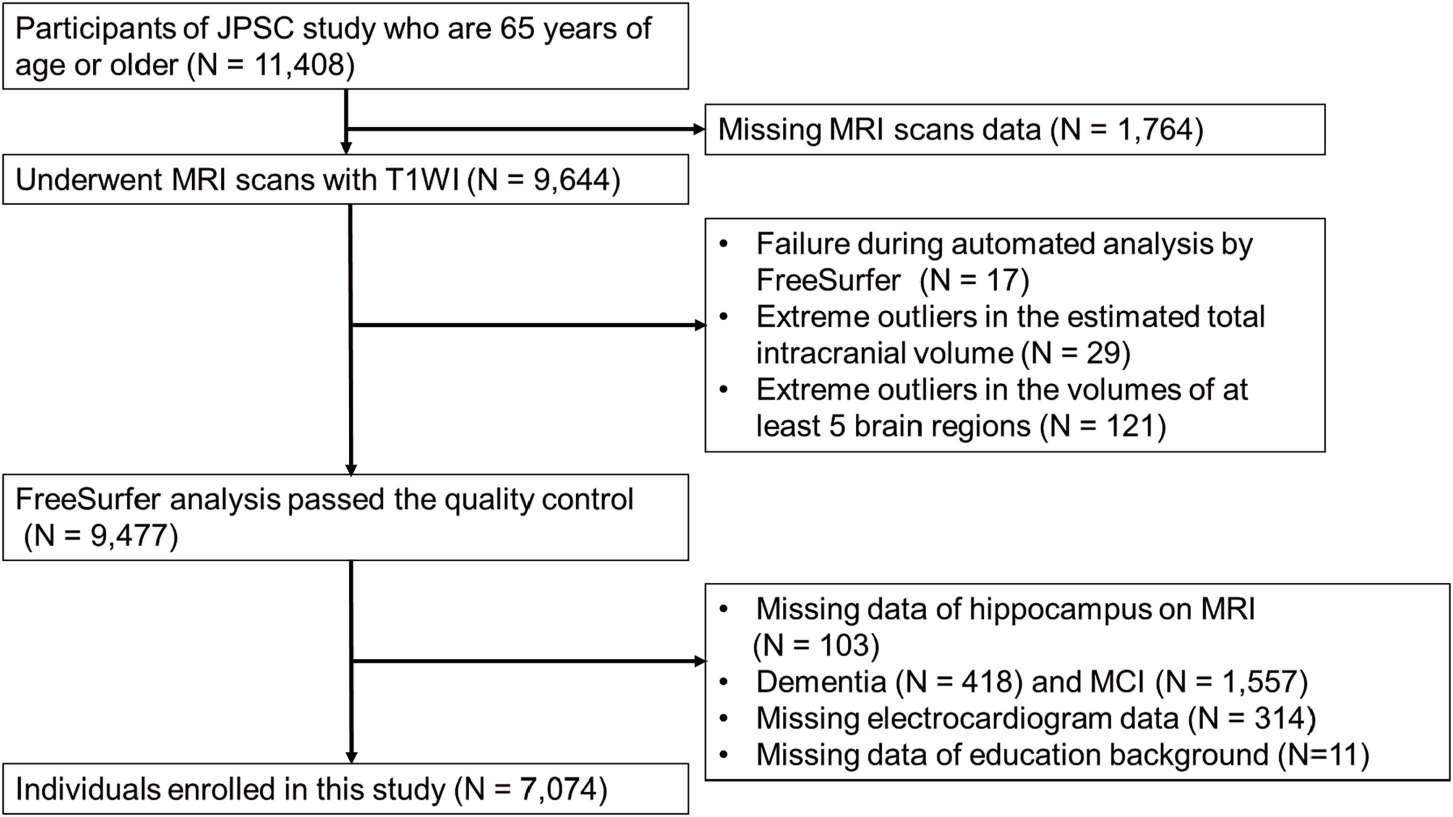
Flowchart of participant selection for the analysis of association between atrial fibrillation/atrial flutter and deep gray matter volumes. JPSC-AD, Japan Prospective Studies Collaboration for Aging and Dementia; MCI, mild cognitive impairment; MRI, magnetic resonance imaging; T1WI, T1-weighted imaging.

We conducted a cross-sectional MRI study in community-dwelling older adults. The baseline survey was conducted between 2016 and 2018. A total of 11,955 community residents from eight research sites consented to participate, of whom 11,408 were aged ≥ 65 years. The detailed study design and methodology have been described previously (Ninomiya et al., 2020). Among these 11,408 participants, 9,644 underwent MRI scans with Three-dimensional (3D) T1-weighted imaging. We excluded 167 participants who failed FreeSurfer quality control, 103 without hippocampal measurement data, 1,975 diagnosed with dementia or mild cognitive impairment, 314 participants without electrocardiogram (ECG) data, and 11 without educational data. The final analytical sample consisted of 7,074 cognitively normal participants (2,893 men and 4,181 women) (Figure 1).

### 2.2 Standard protocol approvals, registrations, and patient consents

The present study was conducted in accordance with the Declaration of Helsinki and was approved through a centralized ethics review by the Kyushu University Institutional Board for Clinical Research (Approval No. 24116). Additional site-specific approvals for study conduct were also obtained from each participating institution. Written informed consent was obtained from all participants.

### 2.3 MRI analysis

Structural MRI was performed using 1.5-T MRI scanners [four Philips, one Hitachi, and one General-Electric (GE)] and 3-T MRI (one GE and one Siemens). Two sites (Arakawa Ward and Hirosaki City) used 3-T MRI; the remaining sites used 1.5-T MRI. 3D T1-weighted turbo field echo images were acquired according to the brain MRI protocol for the Alzheimer’s Disease Neuroimaging Initiative (ADNI) study (Jack et al., 2008). MRI data were standardized using MRI, human, and ADNI phantoms to correct for geometric distortions across scanners.

All T1-weighted images were analyzed using FreeSurfer version 7.0 (FreeSurfer version 7.0^1^) at Tohoku University following standard preprocessing procedures (Fischl et al., 2002; Sämann et al., 2022). FreeSurfer is a widely used and validated automated neuroimaging software for standardized and reproducible volumetric analyses, including hippocampal subfield segmentation in large-scale cohort studies (Sämann et al., 2022). Automated segmentation was used to calculate volumes of the cerebral cortex, cortical white matter, subcortical gray matter, white matter hypointense region, and eight brain regions: the frontal cortex, parietal cortex, temporal cortex, occipital cortex, other cortical areas including the cingulate cortex and the insular cortex, basal ganglia, limbic subcortical structures (including the hippocampus and amygdala), and the diencephalon (including the thalamus proper and ventral diencephalon). Estimated total intracranial volume (eTIV) was also obtained automatically.

**FIGURE 2:**
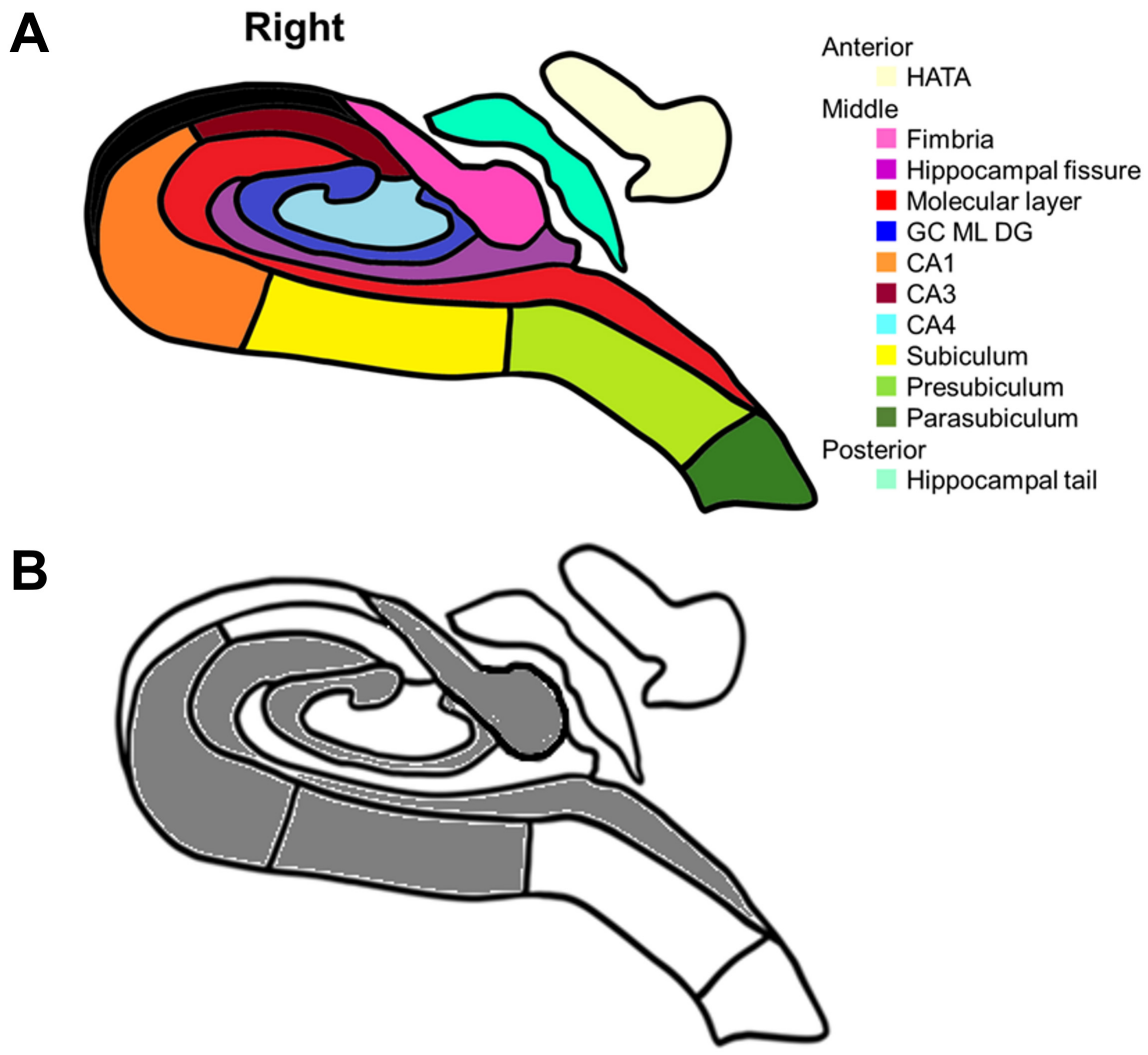
**(A)** Schematic coronal section of the right hippocampus. Hippocampal subfields are represented in different colors. HATA, hippocampus-amygdala-transition-area; GC-DG-ML, granule cell and molecular cell layer of the dentate gyrus; CA, Cornu Ammonis. The HATA subfield is transposed from an anterior coronal section, and the tail is transposed from a posterior coronal section. **(B)** Schematic representation showing substantial associations between hippocampal subregions and atrial fibrillation/atrial flutter status in analysis of the multivariable-adjusted values (Model 2). Gray color represents smaller volumes in the subfields.

Furthermore, among the eight regions, the volumes of the constituent subregions showing significant differences were measured in greater detail. When total hippocampal volume showed significant differences, hippocampal subfield analyses were conducted, including the hippocampal – hippocampus amygdala transition area (HATA), fimbria, hippocampal fissure, molecular layer, granule cell and molecular cell layer of the dentate gyrus (GC ML DG), Cornu Ammonis (CA1, CA3, CA4), subiculum, presubiculum, parasubiculum, and hippocampal tail [Figure 2A, adapted from Figure 2 of Shima et al. (2024), with partial modification].

All regional volumes were calculated as the sum of the right and left hemispheric volumes.

### 2.4 Electrocardiographic data

A standard resting 12-lead ECG was recorded at each site during the baseline health examination. ECG abnormalities, including AF and AFL, were automatically identified and coded according to the Minnesota Code based on the JPSC-AD study protocol (Minnesota Codes 8-3-1 and 8-3-2, respectively). Participants with these ECG findings were classified as having AF/AFL in the present study.

### 2.5 Other risk factors and confounding factors measurements

Potential confounders included age, gender, Apolipoprotein E (*APOE* ε4) status, hypertension, diabetes mellitus (DM), dyslipidemia, smoking, alcohol consumption, low education, and exercise habits. Participant completed a self-administered questionnaire assessing sociodemographic data (age, sex, and educational level), medical history (hypertension, DM, dyslipidemia and heart failure), and lifestyle factors (exercise, smoking, and drinking habits). DM was determined according to the 2010 American Diabetes Association (ADA) criteria (American Diabetes Association, 2010). Regular exercise was defined as engaging in physical activity or sports for at least 30 min twice per week over the previous year or longer. The completed questionnaires were inspected by trained researchers to identify unanswered or inconsistent items. APOE polymorphisms (rs429358 and rs7412) were genotyped using multiplex PCR-based targeted sequencing as previously reported (Momozawa et al., 2016).

### 2.6 Statistical analyses

Clinical characteristics were evaluated using the *t*-test and chi-square test for continuous and categorical variables, respectively. Analysis of covariance was used to estimate and compare multivariable-adjusted values and 95% confidence intervals (CIs). Model 1 was adjusted for age, sex, research site, educational level, and eTIV. Model 2 was further adjusted for the presence of *APOE* ε4 allele, hypertension, DM, dyslipidemia, heart failure, regular exercise, and smoking and drinking habits.

Multiple comparisons were addressed using the Benjamini–Hochberg false discovery rate (FDR) procedure (Benjamini and Hochberg, 1995). FDR-adjusted *p*-values (*q*-values) <0.05 were considered statistically significant. All analyses were conducted using SPSS software (version 29; SPSS Inc., Chicago, IL, USA). In addition to ECG-defined AF/AFL, exploratory analyses using a self-reported history of AF were also performed to examine whether historical AF status showed similar associations with brain volumetric measures. To confirm the interaction between AF/AFL and other risk factors for hippocampal atrophy, stratified analyses by age and sex were performed, and the interaction effect was confirmed. A stratified analysis of age was conducted separately for those under 75 and over 75 years of age.

## 3 Results

### 3.1 Characteristics of participants

Among the 7,074 study participants, 171 (2.4%) were diagnosed with AF/AFL. Table 1 presents the clinical characteristics according to AF/AFL status. Participants with AF/AFL were significantly older and had higher eTIV. They also had higher prevalence of hypertension, heart failure, DM, fewer than 9 years of formal education, smoking, and alcohol consumption. Conversely, the proportion of women with AF/AFL was significantly lower (Table 1).

**TABLE 1:** Clinical characteristics according to atrial fibrillation/atrial flutter status.

	Atrial fibrillation/atrial flutter	
Variables	Absence	Presence
Number of participants	6903	171
Age, years	72.0 (68–75)	75.1 (69–80)*
Women, %	59.9	25.1*
*APOE* ε4, present, %	17.9	13.6
Hypertension, %	71.5	81.1*
Hyperlipidemia, %	45.7	42.9
Diabetes mellitus, %	15.6	24.9*
Heart failure, %	0.7	4*
Education ≤ 9 y, %	24.7	35.7*
Smoking habit, %	8.3	10.5*
Drinking habit, %	44.3	51.5*
Regular exercise, %	44.2	38.9
Estimated intracranial volume, ×10^3^ mm^3^	14.1 (13.0–15.1)	14.8 (13.9–15.9)*

*APOE*, apolipoprotein E. Values were shown as mean values (standard deviations) or frequencies.

*P for trend <0.05.

### 3.2 Associations between regional brain volumes and AF/AFL

We first examined the association between AF/AFL and the volumes of major brain regions, including the cerebral cortex, cortical white matter, and subcortical gray matter. AF/AFL was consistently associated with smaller volumes in all three regions after adjustment for age, sex, research site, educational levels, and eTIV (Model 1), and remained significant after further adjustment for *APOE* ε4 status, hypertension, heart failure, dyslipidemia, diabetes mellitus, smoking, alcohol consumption, and regular exercise (Model 2) (Table 2 and Supplementary Table 1).

**TABLE 2:** Multivariable-adjusted mean values of the volumes of the individual cerebral regions according to atrial fibrillation/atrial flutter status.

	Atrial fibrillation/atrial flutter			
Brain region	Absence (*n* = 6590)	Presence (*n* = 162)	*P* for trend	*Q*-value of FDR correction
Cortex, ×10^4^ mm^3^	38.089 (37.753–38.426)	37.656 (37.199–38.113)	0.01*	–
Cortical white matter, ×10^4^ mm^3^	41.009 (40.581–41.438)	40.422 (39.839–41.005)	0.008*	–
Subcortical gray matter, ×10^3^ mm^3^	48.181 (47.680–48.692)	47.299 (46.611–47.987)	<0.001*	–
White matter hypointense region, ×10^2^ mm^3^	50.695 (43.416–57.974)	55.895 (45.995–65.795)	0.16	–
Each brain region				
Frontal cortex, ×10^4^ mm^3^	13.015 (12.8857–13.144)	12.858 (12.684–13.033)	0.01*	0.048^†^
Parietal cortex, ×10^3^ mm^3^	96.990 (95.967–98.012)	95.940 (94.548–97.333)	0.04*	0.07
Temporal cortex, ×10^2^ mm^3^	44.785 (43.957–45.614)	44.390 (43.263–45.517)	0.35	0.35
Occipital cortex, ×10^3^ mm^3^	36.504 (36.019–36.989)	35.930 (35.270–36.591)	0.02*	0.046^†^
Other cortical area, ×10^3^ mm^3^	28.836 (28.514–29.157)	28.575 (28.138–29.012)	0.11	0.15
Basal ganglia, ×10^3^ mm^3^	18.319 (18.008–18.630)	18.097 (17.676–18.519)	0.16	0.18
Limbic subcortical structures, ×10^2^ mm^3^	88.271 (87.102–89.439)	86.542 (84.952–88.131)	0.004*	0.032^†^
Diencephalon, ×10^3^ mm^3^	18.503 (18.267–18.740)	18.202 (17.881–18.524)	0.01*	0.056
Limbic subcortical structures				
Hippocampus, ×10^2^ mm^3^	61.967 (61.167–62.775)	60.840 (59.746–61.934)	0.007*	–
Amygdala, ×10^2^ mm^3^	26.298 (25.776–26.819)	25.698 (24.989–26.407)	0.02*	–

eTIV, estimated intracranial volume; FDR, false discovery rate. Each regional brain volume was calculated as the sum of the left and right volumes. Values are shown as multivariable-adjusted mean values (95% confidence intervals) after adjusting for age, sex, educational level, research site, apolipoprotein E *ε4*, hypertension, dyslipidemia, diabetes mellitus, heart failure, current smoking habits, current alcohol intakes, regular exercise, and eTIV.

*P for trend < 0.05.

† *Q*-value of FDR correction < 0.05.

We then analyzed eight regions: the frontal cortex, parietal cortex, temporal cortex, occipital cortex, other cortical are (including the cingulate cortex and the insular cortex), basal ganglia, limbic subcortical structures (including the hippocampus and amygdala), and diencephalon (including the thalamus proper and ventral diencephalon). In Model 1, substantial differences were observed in the frontal, parietal, and occipital cortices, as well as in the limbic subcortical structures, and diencephalon (Supplementary Table 1). After full adjustment (Model 2), significant differences persisted in the frontal cortex, occipital cortex, and limbic subcortical structures (Table 2).

Further analyses of limbic subcortical structures revealed that the hippocampus, amygdala showed consistently smaller volumes in participants with AF/AFL in both Model 1 (Supplementary Table 1) and Model 2 (Table 2).

### 3.3 Associations of hippocampal subfield volumes with AF/AFL

We next examined 12 hippocampal subfields: hippocampal amygdala transition area (HATA), fimbria, hippocampal fissure, molecular layer, granule cell and molecular cell layer of the dentate gyrus (GC ML DG), Cornu Ammonis (CA1, CA3, CA4), subiculum, presubiculum, parasubiculum, and hippocampal tail (Figure 2A). The distributions of outcome variables such as subfield volumes were approximately normal. Figure 2B shows the hippocampal subfields with significantly lower volumes in the AF/AFL group. In both Model 1 and Model 2, substantial differences were observed in the fimbria, molecular layer, GC ML DG, CA1, and subiculum (Figure 2B, Table 3 and Supplementary Table 1).

**TABLE 3:** Multivariable-adjusted mean values of the volumes of the hippocampal subfields according to atrial fibrillation/atrial flutter status.

	Atrial fibrillation/atrial flutter			
Brain regions	Absence (*n* = 6590)	Presence (*n* = 162)	*P* for trend	*Q*-value of FDR correction
HATA, HV, mm^3^	96.976 (94.635–99.318)	95.361 (92.176–98.545)	0.18	0.24
Fimbria, HV, mm^3^	101.364 (96.326–106.401)	94.692 (87.840–101.543)	0.011*	0.044^†^
Hippocampal fissure, HV, mm^3^	347.997 (340.453–355.541)	345.139 (334.878–355.400)	0.46	0.46
Molecular_layer, HV, mm^3^	805.311 (792.468–818.155)	786.191 (768.721–803.660)	0.004*	0.048^†^
GC ML DG, HV, mm^3^	540.102 (531.835–548.396)	528.599 (517.355–539.844)	0.007*	0.042^†^
CA1, HV, mm^3^	1217.357 (1198.283–1236.430)	1192.690 (1166.746–1218.633)	0.013*	0.039^†^
CA3, HV, mm^3^	396.000 (388.174–403.827)	389.132 (378.487–399.777)	0.09	0.15
CA4, HV, mm^3^	478.107 (470.882–485.333)	470.304 (460.476–480.131)	0.038*	0.07
Subiculum, HV, mm^3^	836.994 (823.798–850.190)	820.186 (802.237–838.135)	0.014*	0.033^†^
Presubiculum, HV, mm^3^	563.739 (553.218–574.261)	559.098 (544.787–573.409)	0.39	0.42
Parasubiculum, HV, mm^3^	110.943 (107.106–114.780)	112.894 (107.675–118.113)	0.32	0.38
Hippocampal tail, HV, mm^3^	1050.254 (1030.114–1070.395)	1034.920 (1007.526–1062.315)	0.14	0.21

CA, Cornu Ammonis; eTIV, estimated intracranial volume; FDR, false discovery rate; GC ML DG, granule cell and molecular cell layer of the dentate gyrus; HATA, hippocampus amygdala transition area; HV, hippocampal volume. Each regional brain volume was calculated as the sum of the left and right volumes. Values are shown as multivariable-adjusted mean values (95% confidence intervals) after adjusting for age, sex, educational level, research site, apolipoprotein E *ε4*, hypertension, dyslipidemia, diabetes mellitus, heart failure, current smoking habits, current alcohol intakes, regular exercise, and eTIV.

*P for trend < 0.05.

† *Q*-value of FDR correction < 0.05.

### 3.4 Subgroup analyses by history of atrial fibrillation, age and sex

In exploratory analyses using a self-reported history of AF as the exposure variable, no significant associations with regional brain or hippocampal subfield volumes were observed after multivariable adjustment (Supplementary Tables 2, 3).

In age-stratified analyses, significant associations between AF/AFL and hippocampal volume were observed only among participants younger than 75 years of age. Within this group, GC ML DG and CA4 volumes were significantly smaller. Among the hippocampal subfields, only the hippocampal tail showed a statistically significant interaction effect with age (Supplementary Table 4).

In sex-stratified analyses, women showed significant differences in the hippocampus; however, no significant interaction terms were observed (Supplementary Table 5).

## 4 Discussion

This cross-sectional study demonstrated that AF/AFL was associated with smaller volumes in major brain regions, including the cortex, cortical white matter, and subcortical gray matter, as well as in the hippocampus, amygdala. At the hippocampal subfield level, AF/AFL was associated with reduced fimbria, molecular layer, GC ML DG, CA1, and subiculum volumes. To our knowledge, this is the first large-scale study to examine association between AF/AFL and volume alterations in deep gray matter structures and hippocampal subfields in cognitively normal individuals. In age-stratified analyses, significant associations between AF/AFL and hippocampal volume were observed only among participants younger than 75 years of age. Within this group, GC ML DG and CA4 volumes were significantly smaller, while hippocampal tail volume showed a nominal reduction with a significant interaction effect for age. In sex-stratified analyses, women showed smaller hippocampal volumes; however, no significant interaction effects were observed. In addition, exploratory analyses using a self-reported history of AF did not demonstrate significant associations with regional brain or hippocampal subfield volumes. These findings suggest that structural vulnerability associated with AF/AFL may be detectable in specific subregions and demographic subgroups prior to clinically detectable cognitive impairment. Importantly, the hippocampus comprises multiple subfields with distinct cytoarchitecture, connectivity, and vascular supply, and these subregions differ in their vulnerability to ischemia, hypoperfusion, and neurodegenerative processes. Analyses restricted to total hippocampal volume may therefore obscure region-specific structural alterations. By examining hippocampal subfields, the present study was able to identify selective volume reductions in the fimbria, molecular layer, dentate gyrus, CA1, and subiculum, suggesting that AF/AFL-related brain alterations may preferentially affect specific hippocampal circuits. These findings provide more detailed neuroanatomical insights into the potential mechanisms underlying AF/AFL-associated brain vulnerability.

Previous studies have reported that AF is associated with reduced total brain volume (Stefansdottir et al., 2013) and decreased cortical, white matter, and deep subcortical volumes (Moazzami et al., 2020), particularly in persistent AF and with longer AF duration (Stefansdottir et al., 2013). Regional cortical volume changes have been inconsistent, with some studies identifying frontal cortical atrophy (Piers et al., 2016), and others reporting parietal, temporal, or occipital changes (Moazzami et al., 2020). In our study, significant volume reductions were observed in the frontal and occipital cortices after FDR correction, in addition to alterations in subcortical and limbic structures, suggesting that AF/AFL-related brain vulnerability may involve both cortical and subcortical regions.

While several studies have reported smaller hippocampal volumes in AF (Lappegård et al., 2013; Moazzami et al., 2020; Stefan et al., 2008; Stefansdottir et al., 2013), others have reported no significant association when AF is treated as a single exposure (Graff-Radford et al., 2016; Piers et al., 2016). Notably, none have further evaluated hippocampal subfield volume in relation to AF. Our findings extend the literature by demonstrating selective vulnerability of the fimbria, CA1, subiculum, and the dentate gyrus. Reduced amygdala volume has been described in AF (Lappegård et al., 2013). Patients with AF have reported impairments in learning, memory, attention, and executive function (Stefan et al., 2008; Stefansdottir et al., 2013), with a more pronounced decline in patients with persistent AF (Stefan et al., 2008). In addition, in patients with Alzheimer’s disease and amnestic mild cognitive impairment, AF has been associated with more rapid cognitive decline and greater white matter lesion burden, further supporting the notion that AF may exacerbate neurodegenerative processes beyond clinically overt stroke (Nakase et al., 2023). It remains unclear whether AFL alone confers similar cognitive effects to AF (Saglietto et al., 2022). Because AFL is commonly analyzed together with AF, its independent contribution to brain volume also warrants further investigation. Taken together, our findings support and extend prior evidence indicating that AF is associated with structural alterations in brain regions crucial for memory, attention, and emotional processing while additionally revealing subregional hippocampal vulnerability, which has not been previously identified.

The hippocampus and amygdala are key components of memory and cognitive networks that support episodic memory, attention, and emotional processing (Dolcos et al., 2011; Phelps, 2004; Sherman, 2016; Van der Werf et al., 2002). Structural alterations in these regions may therefore contribute to multidomain cognitive vulnerability. Within the hippocampus, individual subfields play distinct roles in memory processing. CA1 and the subiculum are critically involved in episodic and spatial memory (Bartsch et al., 2011; Jeong et al., 2018; Matsumoto et al., 2019; Morris et al., 1990; Tamura et al., 2000), whereas the dentate gyrus contributes to novelty detection, pattern separation, and memory consolidation (Kitchigina et al., 2022; Sasaki et al., 2018; van Dijk and Fenton, 2018). In addition, the fimbria contains myelinated hippocampal projection fibers involved in memory-related neural circuits, including the Papez circuit, and structural alterations in this region may therefore reflect impaired hippocampal connectivity associated with AF/AFL-related white matter vulnerability (Dahmani et al., 2020). Selective structural alterations in these subfields may therefore represent early deficits in memory organization and contextual integration, even in cognitively normal individuals.

The selective involvement of specific hippocampal subfields observed in the present study may be particularly informative because different subfields exhibit distinct molecular characteristics, vascular supply, and susceptibility to ischemic or inflammatory injury. AF has been hypothesized to affect the brain through multiple interacting pathophysiological mechanisms, including (1) microembolic events and silent infarction, (2) hemodynamic stress due to cerebral hypoperfusion and beat-to-beat variability in cardiac output, (3) small-vessel disease and cerebral microbleeds, and (4) systemic inflammation, neuroinflammation, and oxidative stress (Agarwal et al., 2024). These mechanisms likely contribute cumulatively to chronic brain injury. Numerous epidemiological studies and meta-analyses have demonstrated that AF is associated with an increased risk of cognitive impairment and dementia irrespective of clinically overt stroke (Koh et al., 2022; Papanastasiou et al., 2021), suggesting that cognitive dysfunction in AF reflects a multifactorial interplay. In patients with AF, silent cerebral infarcts, white matter lesions, small-vessel disease, and cerebral microbleeds are frequently observed and have been associated with cognitive decline comparable to that observed in clinical brain infarction (Agarwal et al., 2024; Koh et al., 2022; Papanastasiou et al., 2021), reflecting recurrent microembolic events and chronic cerebral hypoperfusion that promote demyelination and microvascular injury. In addition, AF-related beat-to-beat variability in cardiac output may impair cerebrovascular autoregulation and induce microcirculatory hemodynamic stress, and contributes to the progression of white-matter pathology and cognitive decline (Agarwal et al., 2024; Koh et al., 2022; Papanastasiou et al., 2021). Elevated inflammatory cytokine and C-reactive protein levels in AF indicate a heightened systemic inflammatory response that promotes endothelial dysfunction, disruption of the blood–brain barrier, neuroinflammation, and oxidative stress, leading to neuronal and glial injury and degeneration (Agarwal et al., 2024; Lappegård et al., 2013). Interestingly, exploratory analyses using self-reported history of AF did not demonstrate significant associations with brain or hippocampal subfield volumes, whereas ECG-defined AF/AFL showed significant associations. This discrepancy may suggest that active or ongoing arrhythmia-related hemodynamic instability, rather than a remote history of AF alone, may be more closely associated with structural brain vulnerability. Alternatively, self-reported history may have been subject to misclassification or may have included participants with adequately controlled or transient arrhythmia burden. Prospective studies incorporating continuous rhythm monitoring may help clarify the relationship between arrhythmia burden and structural brain alterations.

In contrast, the effects of AFL on brain tissue and cerebral perfusion remain unclear. As AFL typically exhibits a more regular rhythm than AF, fluctuations in cardiac output and cerebral blood flow are less pronounced. To our knowledge, no prior study has evaluated AFL-specific cerebral perfusion. AFL has been reported to be associated with a higher proportion of lacunar-type infarctions than AF (Staszewski et al., 2019), possibly reflecting higher prevalence of vascular risk factors, such as hypertension, smoking, obesity, and dyslipidemia. These factors are closely related to cerebral small-vessel disease and penetrating arteriolar pathology, and they may partly account for the greater frequency of small deep infarcts. Although the hippocampus is not a typical site for lacunar infarction, hippocampal pathology may occur through the occlusion of small branches of the posterior cerebral artery or microinfarcts associated with small-vessel disease.

The volumetric reductions associated with AF/AFL were observed in the CA1, the fimbria, the subiculum, and the dentate gyrus. These selective vulnerabilities may reflect the distinct biological and vascular characteristics of hippocampal subfields.

Cornu Ammonis1 pyramidal neurons exhibit a high density of glutamatergic NMDA receptors, and ischemia-related glutamatergic overactivation induces intracellular Ca^2+^ influx, triggering excitotoxic cascades that lead to mitochondrial dysfunction and neuronal death (Bartsch et al., 2015). These features render CA1 particularly susceptible to ischemic injury.

Regarding the fimbria, because it consists predominantly of myelinated hippocampal projection fibers, it may be vulnerable to chronic hypoperfusion and white matter injury associated with AF/AFL (Agarwal et al., 2024; Koh et al., 2022; Papanastasiou et al., 2021). The subiculum is supplied by small superficial hippocampal arteries with limited collateral circulation, which may increase its vulnerability to chronic hypoperfusion and microvascular injury (Duvernoy et al., 2013). The dentate gyrus, a principal site of adult hippocampal neurogenesis, may be particularly sensitive to inflammatory and metabolic stress that can impair neurogenic activity (Kohman and Rhodes, 2013; Krisai et al., 2025; Liu et al., 2014). Together, these characteristics suggest that AF/AFL-related hemodynamic instability and inflammation may preferentially affect specific hippocampal subregions. The amygdala volume reduction observed in this study may reflect a combined vascular and inflammatory vulnerability. The amygdala is supplied by small cortical and perforating branches from the anterior choroidal and posterior cerebral arteries with limited collateralization (Javed and Das, 2023; Yu et al., 2018), rendering them vulnerable to hypoperfusion and microischemic injury. Experimental studies have also demonstrated glial remodeling and pro-inflammatory activation within the amygdala under chronic cardiovascular stress (Althammer et al., 2020), supporting its susceptibility to systemic and neuroinflammatory processes. Therefore, hemodynamic instability or microembolism may result in insufficient compensatory flow, rendering these territories vulnerable to chronic hypoperfusion. Taken together, these findings suggest that AF/AFL-related injuries may emerge early in perfusion-sensitive regions and accumulate progressively, even in cognitively normal individuals.

Although hippocampal tail volume did not remain statistically significant after multiple-comparison correction in the age-stratified analysis, the significant interaction effect with age may suggest possible regional vulnerability of the posterior hippocampus. The hippocampal tail is supplied by distal branches of the posterior cerebral artery and may be susceptible to hemodynamic stress and chronic hypoperfusion because of the relatively vulnerable microvascular architecture of the hippocampus (Spallazzi et al., 2018). However, these findings should be interpreted cautiously and require confirmation in future studies.

The present findings may have important clinical implications because hippocampal subfields are functionally and biologically heterogeneous and exhibit differential vulnerability to ischemia, hypoperfusion, inflammation, and neurodegenerative processes. Analyses restricted to total hippocampal volume may therefore overlook selective structural alterations associated with AF/AFL. In the present study, selective involvement of the fimbria, dentate gyrus, CA1, and subiculum suggests that AF/AFL-related brain injury may preferentially affect specific hippocampal circuits involved in memory processing and cognitive integration. Importantly, these structural alterations were observed even in cognitively normal individuals, raising the possibility that hippocampal subfield analysis may help identify early and region-specific brain vulnerability before clinically overt cognitive impairment becomes apparent.

These findings further suggest that early cardiovascular risk management and rhythm-control strategies in AF/AFL may have implications beyond stroke prevention. If AF/AFL-related hemodynamic instability, inflammation, and microvascular injury contribute to selective hippocampal vulnerability, earlier intervention may potentially help preserve brain structural integrity and cognitive function. In this context, hippocampal subfield alterations may serve as potential imaging biomarkers for identifying individuals at increased risk of future cognitive decline or dementia. However, prospective longitudinal studies are needed to determine whether therapeutic intervention can modify these structural brain changes.

A major strength of this study is its large sample size, which enabled the adjustment for numerous potential confounders. However, several limitations should be acknowledged. First, because cross-sectional design precludes causal inference. Second, information on AF/AFL duration and subtype (persistent or paroxysmal) was not available. In addition, AF and AFL were coded together according to the study protocol, and separate classification of AF and AFL was unavailable; therefore, we could not independently analyze AF and AFL or determine whether the observed associations were primarily driven by either arrhythmia subtype. Detailed information regarding anticoagulant therapy was also unavailable for the present analysis, and thus we could not evaluate the potential influence of anticoagulation status on the association between AF/AFL and brain volumetric changes. AF/AFL was also identified based on a single electrocardiographic recording; therefore, paroxysmal atrial fibrillation may have been underdetected. Third, MRI scanners differed across sites (1.5-T vs. 3-T), and although standardization procedures and statistical adjustments were applied, residual variability may remain. Finally, all participants were Japanese, which may limit generalizability.

In conclusion, AF/AFL was associated with reduced volumes of the hippocampus and amygdala, as well as selective alterations in hippocampal subfields, in cognitively normal individuals. These findings raise the possibility that AF/AFL-related structural brain changes may occur before clinically detectable cognitive impairment. Further prospective longitudinal studies are needed to clarify whether early cardiovascular and rhythm-control interventions can modify these structural brain alterations and subsequent cognitive outcomes.

## Data Availability

The datasets presented in this article are not publicly available because they contain confidential clinical data from study participants. Requests to access the datasets should be directed to the Principal Investigator, Toshiharu Ninomiya, and will be considered upon reasonable request and subject to institutional and ethical approval.

## Footnotes

1
http://surfer.nmr.mgh.harvard.edu
