# Supplementary material for: Atrial fibrillation/atrial flutter and reduced volumes of the hippocampus, amygdala, and thalamus: evidence from a community-based MRI study
Source: Front Aging Neurosci. 2026 Jul 17;18:1848538. doi: 10.3389/fnagi.2026.1848538 (PMC13423846; doi:10.3389/fnagi.2026.1848538)
Supplement: Supplementary file 1 [file Data_Sheet_1.docx]

| **Supplementary Table 1. Mean values of the volumes of the individual brain areas and hippocampal subregions according to atrial fibrillation/atrial flutter in model 1** | | | | |
| --- | --- | --- | --- | --- |
|  | Atrial fibrillation/atrial flutter | | p for trend | q-value of FDR correction |
|  | Absence  (n=6,903) | Presence  (n=171) |  |  |
| Cortex, ×10^4^ mm^3^ | 38.021 (37.869-38.173) | 37.535 (37.179-37.891) | 0.005* | - |
| Cortical white matter, ×10^4^ mm^3^ | 41.340 (41.148-41.532) | 40.742 (40.291-41.193) | 0.006* | - |
| Subcortical gray matter, ×10^3^ mm^3^ | 48.764 (48.536-48.992) | 48.000 (47.466-48.535) | 0.003* | - |
| White matter hypointense region, ×10^2^ mm^3^ | 50.895 (47.605-54.184) | 56.784 (49.064-64.503) | 0.11 | - |
| ***Individual brain regions*** |  |  |  |  |
| Frontal cortex, ×10^4^ mm^3^ | 12.984 (12.927-13.042) | 12.795 (12.660-12.930) | 0.004* | 0.02^†^ |
| Parietal cortex, ×10^3^ mm^3^ | 97.129 (96.669-97.589) | 95.953 (94.878-97.029) | 0.02* | 0.04^†^ |
| Temporal cortex, ×10^2^ mm^3^ | 45.081 (44.709-45.453) | 44.544 (43.670-45.417) | 0.20 | 0.23 |
| Occipital cortex, ×10^3^ mm^3^ | 36.506 (36.289-36.723) | 35.909 (35.399-36.420) | 0.02* | 0.03^†^ |
| Other cortical area, ×10^3^ mm^3^ | 28.787 (28.642-28.932) | 28.491 (28.151-28.832) | 0.07 | 0.09 |
| Basal ganglia, ×10^3^ mm^3^ | 18.794 (18.655-18.934) | 18.673 (18.346-19.0000) | 0.44 | 0.44 |
| Limbic subcortical structures, ×10^2^ mm^3^ | 87.917 (87.393-88.442) | 86.156 (84.926-87.387) | 0.003* | 0.02^†^ |
| Diencephalon, ×10^3^ mm^3^ | 18.738 (18.633-18.844) | 18.424 (18.176-18.672) | 0.008* | 0.02^†^ |
| ***Limbic subcortical structure*** |  |  |  |  |
| Hippocampus, HV, ×10^2^ mm^3^ | 61.968 (61.607-62.329) | 60.784 (59.937-61.632) | 0.004* | 0.02^†^ |
| Amygdala, ×10^2^ mm^3^ | 25.949 (25.716-56.182) | 25.369 (24.823-25.916) | 0.03* | 0.04^†^ |
| ***Diencephalon*** |  |  |  |  |
| Thalamus_proper, ×10^3^ mm^3^ | 12.200 (12.110-12.289) | 11.943 (11.733-12.153) | 0.01* | 0.02^†^ |
| Ventral diencephalon, ×10^2^ mm^3^ | 10.553 (10.462-10.643) | 10.385 (10.172-10.597) | 0.10 | 0.10 |
| ***Hippocampal subregions*** |  |  |  |  |
| HATA, HV, mm^3^ | 97.246 (96.195-98.296) | 95.253 (92.789-97.718) | 0.09 | 0.14 |
| Fimbria, HV, mm^3^ | 101.633 (99.373-103.893) | 95.022 (89.718-100.327) | 0.01* | 0.02^†^ |
| Hippocampal fissure, HV, mm^3^ | 348.439 (345.060-351.818) | 345.059 (337.129-352.988) | 0.38 | 0.38 |
| Molecular_layer, HV, mm^3^ | 805.454 (799.697-811.211) | 786.233 (772.722-799.745) | 0.003* | 0.04^†^ |
| GC ML DG, HV, mm^3^ | 540.896 (537.191-544.601) | 529.076 (520.381-537.771) | 0.005* | 0.03^†^ |
| CA1, HV, mm^3^ | 1213.275 (1204.739-1221.812) | 1187.908 (1167.874-1207.942) | 0.008* | 0.03^†^ |
| CA3, HV, mm^3^ | 398.786 (395.284-402.288) | 391.119 (382.900-399.337) | 0.05 | 0.09 |
| CA4, HV, mm^3^ | 478.840 (475.606-482.074) | 470.692 (463.102-478.281) | 0.03* | 0.05 |
| Subiculum, HV, mm^3^ | 838.194 (832.282-844.106) | 820.417 (806.542-834.293) | 0.008* | 0.03^†^ |
| Presubiculum, HV, mm^3^ | 558.478 (553.763-563.193) | 553.738 (542.673-564.803) | 0.37 | 0.41 |
| Parasubiculum, HV, mm^3^ | 108.694 (106.972-110.415) | 110.487 (106.447-114.527) | 0.34 | 0.40 |
| Hippocampal tail, HV, mm^3^ | 1055.341 (1046.285-1064.397) | 1038.541 (1017.286-1059.795) | 0.10 | 0.13 |
| Abbreviations: CA, Cornu Ammonis; eTIV, estimated intracranial volume; FDR, false discovery rate; GC ML DG, granule cell and molecular cell layer of the dentate gyrus; HATA, hippocampus amygdala transition area; HV, hippocampal volume.  Values are shown as multivariable-adjusted mean values (95% confidence intervals), where values are calculated as follows: (left + right) volumes of individual brain areas or hippocampal subregions. Adjusted for age, sex, educational level, research site, eTIV.  * p for trend < 0.05. † q-value of FDR correction < 0.05. | | | | |

| **Supplementary Table 2. Multivariable-adjusted mean values of the volumes of the individual cerebral regions according to** **history of atrial fibrillation** | | | | |
| --- | --- | --- | --- | --- |
| Brain region | History of atrial fibrillation | | p for trend | q-value of FDR correction |
|  | Absence  (n=6,424) | Presence  (n=327) |  |  |
| Cortex, ×10^4^ mm^3^ | 38.071 (37.735-38.408) | 37.897 (37.500-38.294) | 0.15 | - |
| Cortical white matter, ×10^4^ mm^3^ | 40.986 (40.557-41.414) | 40.741 (40.235-41.247) | 0.12 | - |
| Subcortical gray matter, ×10^3^ mm^3^ | 48.119 (47.612-48.625) | 48.014 (47.416-48.612) | 0.57 | - |
| White matter hypointense region, ×10^2^ mm^3^ | 51.398 (44.122-58.675) | 49.394 (40.800-58.675) | 0.45 | - |
| *Each brain region* |  |  |  |  |
| Frontal cortex, ×10^4^ mm^3^ | 13.012 (12.883-13.140) | 12.916 (12.764-13.068) | 0.04* | 0.34 |
| Parietal cortex, ×10^3^ mm^3^ | 96.963 (95.941-97.985) | 96.436 (95.227-97.644) | 0.15 | 0.42 |
| Temporal cortex, ×10^2^ mm^3^ | 44.733 (43.904-45.561) | 44.876 (43.898-45.854) | 0.63 | 0.63 |
| Occipital cortex, ×10^3^ mm^3^ | 36.488 (36.003-36.973) | 36.187 (35.614-36.759) | 0.09 | 0.36 |
| Other cortical area, ×10^3^ mm^3^ | 28.826 (28.504-29.147) | 28.712 (28.333-29.091) | 0.33 | 0.53 |
| Basal ganglia, ×10^3^ mm^3^ | 18.289 (17.978-18.600) | 18.659 (18.000-18.732) | 0.49 | 0.66 |
| Limbic subcortical structures, ×10^2^ mm^3^ | 88.142 (86.974-89.311) | 87.909 (86.528-89.289) | 0.58 | 0.66 |
| Diencephalon, ×10^3^ mm^3^ | 18.488 (18.252-18.725) | 18.387 (18.108-18.359) | 0.24 | 0.49 |
| *Limbic subcortical structures* |  |  |  |  |
| Hippocampus, ×10^2^ mm^3^ | 61.897 (61.093-62.701) | 61.665 (60.715-62.615) | 0.43 | - |
| Amygdala, ×10^2^ mm^3^ | 26.244 (25.723-26.766) | 26.239 (25.624-26.855) | 0.98 | - |
| Abbreviations: eTIV, estimated intracranial volume; FDR, false discovery rate  Each regional brain volume was calculated as the sum of the left and right volumes.  Values are shown as multivariable-adjusted mean values (95% confidence intervals) after adjusting for age, sex, educational level, research site, apolipoprotein E *Ɛ4*, hypertension, dyslipidemia, diabetes mellitus, heart failure, current smoking habits, current alcohol intakes, regular exercise, and eTIV.  ∗ p for trend < 0.05  † q-value of FDR correction < 0.05 | | | | |

| **Supplementary Table 3. Multivariable-adjusted mean values of the volumes of the hippocampal subfields according to history of atrial fibrillation** | | | | |
| --- | --- | --- | --- | --- |
| Brain regions | History of atrial fibrillation | | p for trend | q-value of FDR correction |
|  | Absence  (n=6,424) | Presence  (n=327) |  |  |
| HATA, HV, mm^3^ | 96.899 (94.559-99.239) | 96.315 (93.550-99.079) | 0.49 | 1.19 |
| Fimbria, HV, mm^3^ | 101.034 (95.997-106.070) | 98.764 (92.815-104.713) | 0.22 | 2.65 |
| Hippocampal fissure, HV, mm^3^ | 347.559 (340.018-355.100) | 349.034 (340.127-357.941) | 0.59 | 1.19 |
| Molecular_layer, HV, mm^3^ | 803.503 (790.659-816.348) | 804.167 (788.995-819.339) | 0.88 | 0.96 |
| GC ML DG, HV, mm^3^ | 539.251 (530.983-547.519) | 537.718 (527.953-547.484) | 0.61 | 1.05 |
| CA1, HV, mm^3^ | 1215.511 (1196.441-1234.582) | 1212.276 (1189.750-1234.802) | 0.64 | 0.96 |
| CA3, HV, mm^3^ | 395.375 (387.550-403.200) | 395.420 (386.178-404.662) | 0.98 | 0.98 |
| CA4, HV, mm^3^ | 477.494 (470.269-484.718) | 476.745 (468.212-485.278) | 0.77 | 0.93 |
| Subiculum, HV, mm^3^ | 836.130 (822.935-849.324) | 830.704 (815.119-846.289) | 0.26 | 1.58 |
| Presubiculum, HV, mm^3^ | 563.733 (553.216-574.251) | 560.327 (547.903-572.750) | 0.37 | 1.13 |
| Parasubiculum, HV, mm^3^ | 111.055 (107.219-114.891) | 111.579 (107.048-116.109) | 0.71 | 0.94 |
| Hippocampal tail, HV, mm^3^ | 1049.734 (1029.601-1069.867) | 1042.516 (1018.736-1066.297) | 0.33 | 1.32 |
| Abbreviations: CA, Cornu Ammonis; eTIV, estimated intracranial volume; FDR, false discovery rate; GC ML DG, granule cell and molecular cell layer of the dentate gyrus; HATA, hippocampus amygdala transition area; HV, hippocampal volume  Each regional brain volume was calculated as the sum of the left and right volumes.  Values are shown as multivariable-adjusted mean values (95% confidence intervals) after adjusting for age, sex, educational level, research site, apolipoprotein E *Ɛ4*, hypertension, dyslipidemia, diabetes mellitus, heart failure, current smoking habits, current alcohol intakes, regular exercise, and eTIV.  ∗ p for trend < 0.05  † q-value of FDR correction < 0.05 | | | | |

| **Supplementary Table 4. Multivariable-adjusted mean values of the volumes of the hippocampus and hippocampal subfields, amygdala according to atrial fibrillation/atrial flutter, stratified by age (<75 and ≥75 years)** | | | | | |
| --- | --- | --- | --- | --- | --- |
| Brain regions | Atrial fibrillation/atrial flutter | | p for trend | q-value of FDR correction | p for interaction  between age groups |
|  | Absence | Presence |  |  |  |
| *Age <75 years old* |  |  |  |  |  |
| Number of participants | 4,650 | 75 |  |  |  |
| Hippocampus, HV, ×10^2^ mm^3^ | 62.128 (60.789-63.467) | 60.425 (58.670-62.179) | 0.005* | - | 0.21 |
| Amygdala, ×10^2^ mm^3^ | 26.368 (25.490-27.246) | 25.672 (24.521-26.822) | 0.08 | - | 0.35 |
| *Hippocampal subfields* |  |  |  |  |  |
| HATA, HV, mm^3^ | 95.797 (91.921-99.672) | 93.117 (88.039-98.196) | 0.12 | 0.18 | 0.84 |
| Fimbria, HV, mm^3^ | 105.862 (97.447-114.278) | 100.708 (89.680-111.736) | 0.17 | 0.22 | 0.48 |
| Hippocampal fissure, HV, mm^3^ | 352.736 (340.350-365.122) | 352.698 (336.466-368.930) | 0.99 | 0.99 | 0.05 |
| Molecular_layer, HV, mm^3^ | 812.798 (791.657-833.939) | 795.437 (767.732-823.142) | 0.07 | 0.13 | 0.96 |
| GC ML DG, HV, mm^3^ | 538.450 (524.852-552.048) | 520.350 (502.530-538.170) | 0.004* | 0.04^†^ | 0.35 |
| CA1, HV, mm^3^ | 1223.763 (1191.762-1255.764) | 1190.759 (1148.822-1232.696) | 0.02* | 0.07 | 0.35 |
| CA3, HV, mm^3^ | 395.022 (382.078-407.967) | 382.938 (365.974-399.902) | 0.04* | 0.09 | 0.25 |
| CA4, HV, mm^3^ | 475.839 (464.021-487.657) | 460.697 (445.210-476.185) | 0.005* | 0.03^†^ | 0.45 |
| Subiculum, HV, mm^3^ | 845.264 (823.428-867.100) | 825.662 (797.046-854.278) | 0.04* | 0.09 | 0.64 |
| Presubiculum, HV, mm^3^ | 563.411 (546.100-580.722) | 554.470 (531.783-577.156) | 0.25 | 0.30 | 0.08 |
| Parasubiculum, HV, mm^3^ | 108.823 (102.755-114.891) | 109.535 (101.583-117.486) | 0.79 | 0.86 | 0.09 |
| Hippocampal tail, HV, mm^3^ | 1047.789 (1013.436-1082.142) | 1008.847 (963.828-1053.867) | 0.01* | 0.056 | 0.020^#^ |
| *Age* ≥*75 years old* |  |  |  |  |  |
| Number of participants | 1,940 | 87 |  |  |  |
| Hippocampus, HV, ×10^2^ mm^3^ | 58.560 (57.296-59.824) | 58.006 (56.420-59.591) | 0.33 | - |  |
| Amygdala, ×10^2^ mm^3^ | 24.994 (24.199-25.782) | 24.481 (23.485-25.477) | 0.15 | - |  |
| *Hippocampal subfields* |  |  |  |  |  |
| HATA, HV, mm^3^ | 92.520 (88.795-96.245) | 91.739 (87.066-96.412) | 0.64 | 0.96 |  |
| Fimbria, HV, mm^3^ | 80.567 (72.690-88.443) | 72.505 (62.624-82.387) | 0.02* | 0.28 |  |
| Hippocampal fissure, HV, mm^3^ | 350.086 (338.008-362.165) | 345.177 (330.024-360.329) | 0.36 | 0.86 |  |
| Molecular_layer, HV, mm^3^ | 753.808 (734.278-773.338) | 734.213 (709.713-758.714) | 0.02* | 0.16 |  |
| GC ML DG, HV, mm^3^ | 509.204 (495.850-522.559) | 504.255 (487.502-521.008) | 0.41 | 0.70 |  |
| CA1, HV, mm^3^ | 1152.165 (1122.611-1181.719) | 1136.466 (1099.391-1173.542) | 0.24 | 0.72 |  |
| CA3, HV, mm^3^ | 379.633 (367.212-392.054) | 379.991 (364.409-395.574) | 0.94 | 1.12 |  |
| CA4, HV, mm^3^ | 456.493 (444.702-468.284) | 456.102 (441.310-470.893) | 0.94 | 0.94 |  |
| Subiculum, HV, mm^3^ | 782.502 (761.395-803.609) | 769.595 (743.115-796.074) | 0.17 | 0.68 |  |
| Presubiculum, HV, mm^3^ | 538.712 (521.660-555.764) | 538.578 (517.186-559.969) | 0.98 | 1.07 |  |
| Parasubiculum, HV, mm^3^ | 113.308 (106.574-120.041) | 116.085 (107.638-124.532) | 0.36 | 0.86 |  |
| Hippocampal tail, HV, mm^3^ | 997.112 (967.519-1026.706) | 1001.082 (963.957-1038.280) | 0.76 | 1.01 |  |
| Abbreviations: CA, Cornu Ammonis; eTIV, estimated intracranial volume; FDR, false discovery rate; GC ML DG, granule cell and molecular cell layer of the dentate gyrus; HATA, hippocampus amygdala transition area; HV, hippocampal volume  Each regional brain volume was calculated as the sum of the left and right volumes.  Values are shown as multivariable-adjusted mean values (95% confidence intervals) after adjusting for age, sex, educational level, research site, apolipoprotein E *Ɛ4*, hypertension, dyslipidemia, diabetes mellitus, heart failure, current smoking habits, current alcohol intakes, regular exercise, and eTIV.  * p for trend < 0.05  † q-value of FDR correction < 0.05  #p for interaction < 0.05 | | | | | |

| **Supplementary Table 5. Multivariable-adjusted mean values of the volumes of the hippocampus and hippocampal subfields, amygdala according to atrial fibrillation/atrial flutter, stratified by sex** | | | | | |
| --- | --- | --- | --- | --- | --- |
| Brain regions | Atrial fibrillation/atrial flutter | | p for trend | q-value of FDR correction | p for interaction  between sexes |
|  | Absence | Presence |  |  |  |
| ***Men*** |  |  |  |  |  |
| Number of participants | 2,646 | 122 |  |  |  |
| Hippocampus, HV, ×10^2^ mm^3^ | 64.904 (63.725-66.082) | 64.150 (62.701-65.600) | 0.13 | - | 0.19 |
| Amygdala, ×10^2^ mm^3^ | 27.520 (27.293-28.840) | 27.520 (26.568-28.471) | 0.10 | - | 0.95 |
| ***Hippocampal subfields*** |  |  |  |  |  |
| HATA, HV, mm^3^ | 101.565 (98.111-105.018) | 100.314 (96.067-104.562) | 0.40 | 0.53 | 0.55 |
| Fimbria, HV, mm^3^ | 106.965 (99.327-114.603) | 100.515 (91.120-109.910) | 0.05 | 0.60 | 0.82 |
| Hippocampal fissure, HV, mm^3^ | 360.642 (349.894-371.390) | 359.518 (346.298-372.738) | 0.80 | 0.88 | 0.68 |
| Molecular_layer, HV, mm^3^ | 839.103 (820.379-857.828) | 823.354 (800.322-846.385) | 0.05 | 0.30 | 0.67 |
| GC ML DG, HV, mm^3^ | 564.663 (552.260-577.066) | 554.643 (539.387-569.898) | 0.06 | 0.24 | 0.71 |
| CA1, HV, mm^3^ | 1273.787 (1245.622-1301.952) | 1258.378 (1223.735-1293.021) | 0.20 | 0.34 | 0.18 |
| CA3, HV, mm^3^ | 410.881 (399.094-422.669) | 404.580 (390.081-419.078) | 0.21 | 0.32 | 0.37 |
| CA4, HV, mm^3^ | 500.108 (489.265-510.952) | 493.496 (480.158-506.834) | 0.15 | 0.37 | 0.81 |
| Subiculum, HV, mm^3^ | 877.904 (858.617-897.192) | 865.615 (841.891-889.338) | 0.13 | 0.41 | 0.28 |
| Presubiculum, HV, mm^3^ | 595.165 (579.539-610.791) | 594.442 (575.223-613.662) | 0.91 | 0.91 | 0.18 |
| Parasubiculum, HV, mm^3^ | 119.868 (113.948-125.788) | 123.420 (116.139-130.702) | 0.16 | 0.32 | 0.08 |
| Hippocampal tail, HV, mm^3^ | 1100.405 (1071.616-1129.194) | 1096.333 (1060.923-1131.743) | 0.74 | 0.89 | 0.038^#^ |
| ***Women*** |  |  |  |  |  |
| Number of participants | 3,944 | 40 |  |  |  |
| Hippocampus, HV, ×10^2^ mm^3^ | 60.151 (58.967-61.334) | 58.034 (56.115-59.953) | 0.008* | - |  |
| Amygdala, ×10^2^ mm^3^ | 24.888 (24.127-25.648) | 24.252 (23.019-25.485) | 0.21 | - |  |
| ***Hippocampal subfields*** |  |  |  |  |  |
| HATA, HV, mm^3^ | 94.297 (90.860-97.735) | 91.478 (85.905-97.051) | 0.22 | 0.33 |  |
| Fimbria, HV, mm^3^ | 98.894 (91.683-106.105) | 92.980 (81.290-104.670) | 0.22 | 0.33 |  |
| Hippocampal fissure, HV, mm^3^ | 339.444 (328.065-350.824) | 332.968 (314.520-351.417) | 0.39 | 0.43 |  |
| Molecular_layer, HV, mm^3^ | 787.971 (778.980-806.963) | 763.056 (732.268-793.844) | 0.05 | 0.15 |  |
| GC ML DG, HV, mm^3^ | 524.900 (512.939-510.175) | 510.175 (490.785-529.565) | 0.06 | 0.16 |  |
| CA1, HV, mm^3^ | 1186.595 (1158.673-1214.518) | 1137.894 (1092.626-1183.162) | 0.01* | 0.12 |  |
| CA3, HV, mm^3^ | 390.044 (378.830-401.258) | 386.462 (368.283-404.642) | 0.63 | 0.63 |  |
| CA4, HV, mm^3^ | 463.873 (453.423-474.324) | 453.883 (436.941-470.825) | 0.15 | 0.26 |  |
| Subiculum, HV, mm^3^ | 810.754 (791.251-830.257) | 781.243 (749.626-812.861) | 0.02* | 0.10 |  |
| Presubiculum, HV, mm^3^ | 539.112 (523.787-554.436) | 522.083 (497.239-546.926) | 0.09 | 0.54 |  |
| Parasubiculum, HV, mm^3^ | 102.299 (96.924-107.674) | 98.552 (89.837-107.266) | 0.30 | 0.36 |  |
| Hippocampal tail, HV, mm^3^ | 1016.376 (986.129-1046.622) | 965.648 (916.613-1014.683) | 0.01* | 0.07 |  |
| Abbreviations: CA, Cornu Ammonis; eTIV, estimated intracranial volume; FDR, false discovery rate; GC ML DG, granule cell and molecular cell layer of the dentate gyrus; HATA, hippocampus amygdala transition area; HV, hippocampal volume  Each regional brain volume was calculated as the sum of the left and right volumes.  Values are shown as multivariable-adjusted mean values (95% confidence intervals) after adjusting for age, educational level, research site, apolipoprotein E *Ɛ4*, hypertension, dyslipidemia, diabetes mellitus, heart failure, current smoking habits, current alcohol intakes, regular exercise, and eTIV.  * p for trend < 0.05  † q-value of FDR correction < 0.05  # p for interaction < 0.05. | | | | | |
